# PARTICIPATION RESTRICTIONS IN ISOLATED CERVICAL DYSTONIA: A CONVERGENT MIXED-METHODS STUDY

**DOI:** 10.2340/jrm.v58.46205

**Published:** 2026-07-09

**Authors:** Søren Bruno ELMGREEN

**Affiliations:** 1 Department of Neurology, Aalborg University Hospital, Aalborg; 2 Department of Clinical Medicine, Aalborg University, Aalborg, Denmark

**Keywords:** International Classification of Functioning, Disability and Health, rehabilitation, torticollis

## Abstract

**Objective:**

To describe participation in adults with isolated cervical dystonia and to explore how restrictions are experienced in everyday life.

**Design:**

Convergent mixed-methods study with case- and theme-level integration through joint displays.

**Subjects/Patients:**

Adults with a self-reported specialist diagnosis of cervical dystonia.

**Methods:**

Respondents completed an online survey including the Utrecht Scale for Evaluation of Rehabilitation–Participation. A purposive subsample completed semi-structured interviews, analysed using reflexive thematic analysis. Quantitative data were summarized with 95% confidence intervals.

**Results:**

108 respondents (73% women; mean age 51.6 [SD 14.1] years; mean disease duration 11.6 [SD 12.8] years) completed the survey. Mean scores indicated low frequency (36.6, 95% CI 34.3–38.8), moderate restrictions (67.6, 64.8–70.3), and low satisfaction (43.3, 40.5–46.1); the productivity domain was the most affected (31.6, 28.7–34.5), with leisure (51.3, 48.4–54.2) and social participation (51.5, 49.4–53.6) less restricted but still constrained. Ten participants were interviewed. Three themes were generated: living with unpredictability; invisibility and contested recognition; and a symptom-focused system in a participation-focused life.

**Conclusion:**

Participation was restricted across everyday domains and shaped by fluctuating symptoms, social recognition, and the structure of available care. Patient-reported participation may be a useful complementary outcome alongside impairment-focused assessment.

## LAY ABSTRACT

Cervical dystonia is a neurological condition that pulls the neck muscles into uncomfortable positions and often causes pain. We wanted to know how this affects daily life beyond the neck itself: working, looking after a home, hobbies, and being with other people. In total, 108 adults with cervical dystonia answered an online survey, and 10 of them shared their experience in an interview. People reported low levels of activity, restrictions in work and leisure, and low satisfaction with how they take part in everyday life. They described symptoms that changed from day to day, a condition that was often invisible to others, and healthcare that focused on injections more than on the wider needs of daily life. Follow-up for cervical dystonia should look beyond the neck.

Cervical dystonia is the most common focal dystonia and is characterized by sustained or intermittent muscle contractions causing abnormal movements, postures, or both (1). Clinical management has traditionally centred on motor symptoms, with botulinum neurotoxin injection constituting first-line symptomatic treatment for most patients (2). This approach is clinically important but does not fully capture the wider disability associated with the condition.

A growing literature shows that cervical dystonia is also associated with pain, fatigue, anxiety, depression, sleep disturbance, and reduced quality of life (3–7). These non-motor features may contribute substantially to disability and, in some patients, may be more closely related to everyday functioning than motor severity (5–7). Earlier rehabilitation-oriented work has similarly indicated that disability is shaped by self-efficacy, pain, anxiety, and fatigue in addition to the motor disorder (7), and recent reviews suggest that adjunctive physiotherapy and multidisciplinary rehabilitation may improve pain, functioning, and quality of life, although the evidence base remains limited (8).

Within the International Classification of Functioning, Disability and Health (ICF), participation refers to involvement in life situations and represents a core dimension of disability conceptually distinct from impairment and activity limitation (9). Two recent qualitative studies have begun to describe activity, stigma, and psychological distress in cervical or neck dystonia (10, 11), and these accounts converge on a portrait of disability that is heavily shaped by context. What is still lacking is a participation-specific quantitative description, and an explicit integration of such quantitative description with qualitative accounts of how participation is produced, constrained, and negotiated in daily life.

The Utrecht Scale for Evaluation of Rehabilitation–Participation (USER-P) was developed to assess both objective and subjective aspects of participation in people with physical disabilities (12). The instrument captures frequency of participation, experienced restrictions, and satisfaction with participation, and is therefore well placed to characterize participation in cervical dystonia at the level of everyday roles rather than impairment alone. Quantitative assessment alone, however, cannot fully explain how participation is sustained or eroded; mixed-methods approaches are useful when the aim is both to describe participation and to understand its lived meaning (13, 14).

The aim of the present study was to describe participation outcomes in adults with isolated cervical dystonia using the USER-P, and to explore how participation restrictions are experienced in everyday life. It was anticipated that participation would be restricted across multiple domains and would be shaped not only by symptoms, but also by contextual and social factors.

## METHODS

### Design

A convergent mixed-methods design was used, combining a cross-sectional online survey with semi-structured qualitative interviews (13, 14). The quantitative and qualitative components had equal priority and were collected in parallel. Following Fetters et al. (28), integration occurred at 3 levels: connecting, in that the interview subsample was nested within the survey cohort and purposively selected using survey-derived characteristics (age, sex, occupational situation, and self-reported symptom burden); merging, through case-level and theme-level joint displays; and reporting, through a woven narrative account. Case-level merging was introduced during revision of the manuscript and was not prespecified in the original analysis plan. The quantitative component described participation using a validated participation measure, and the qualitative component examined how participation restrictions were experienced and managed in everyday life. Reporting followed STROBE for the observational component (15), COREQ for the interview component (16), and the Mixed Methods Reporting in Rehabilitation and Health Sciences (MMR-RHS) guidance for the integrated design (14).

### Participants and recruitment

Adults with isolated cervical dystonia were recruited between March and May 2023 through the Danish Dystonia Patient Association, social media channels related to rare neurological disease, and neurology departments in Denmark. Eligibility criteria were age 18 years or older, a self-reported diagnosis of isolated cervical dystonia made by a movement disorder specialist, and ability to complete a Danish-language questionnaire. Diagnosis was not independently verified against current consensus criteria for isolated cervical dystonia (17). No formal exclusion criteria were applied. Potential participants accessed the survey through an online link. At the end of the survey, respondents could indicate willingness to participate in a qualitative interview. A purposive subsample was selected to ensure variation in age, sex, occupational situation, and self-reported symptom burden.

### Quantitative assessment

Quantitative data were collected and managed using REDCap (Research Electronic Data Capture; Vanderbilt University, Nashville, TN, USA), a secure web-based platform for research data capture (18, 19). Participation was assessed with the USER-P (12), comprising 32 items distributed across 3 subscales: participation frequency, participation restrictions, and satisfaction with participation. Summary scores for each subscale range from 0 to 100, with higher scores indicating more favourable participation outcomes. Domain scores for productivity, leisure, and social participation were derived from the relevant items, as detailed in the USER-P manual. Demographic and clinical information included age, sex, and disease duration. Previously published USER-P data from other diagnostic groups were consulted as contextual background during interpretation only and were not used for formal between-group hypothesis testing.

### Qualitative data collection

Semi-structured interviews were conducted face to face by the author (SBE) between May and June 2023. SBE is a male physician with clinical training in neurology and rehabilitation, with prior interview experience but no pre-existing relationship with participants. The author’s clinical background was disclosed at the start of each interview, and participants were informed that the conversation was an opportunity to describe everyday life, not a clinical encounter. An interview guide was used to explore experiences of participation in work, domestic life, leisure activities, social relationships, and interactions with healthcare and support systems (Appendix S1). Interviews lasted 45 to 75 min and took place in participants’ homes, local cafes, or hospital meeting rooms according to participant preference. Interviews were audio-recorded using a RØDE Wireless GO II microphone system (RØDE Microphones, Sydney, Australia) and transcribed verbatim in ATLAS.ti 23.0.1 (ATLAS.ti Scientific Software Development GmbH, Berlin, Germany).

### Qualitative data analysis

Interviews were analysed using reflexive thematic analysis as articulated by Braun and Clarke (20, 21). Within this framework, themes are understood as patterns of shared meaning generated through the analyst’s active engagement with the data, rather than as topics that emerge independently of the researcher. A single-analyst design is methodologically consistent with reflexive thematic analysis when reflexive practice and an audit trail are maintained (20, 21). Analysis followed the 6 recursive phases: familiarization, generation of initial codes, construction of candidate themes, review against the dataset and study aim, definition and naming of themes, and writing-up. A working codebook and analytic memos were maintained for each participant (Appendix S2). Reflexivity was supported by written memos in which the analyst examined how a clinical neurology perspective shaped early coding, in particular by initially privileging symptom-focused codes; subsequent coding was consciously broadened to contextual and relational dimensions. Sample size was guided by the concept of information power (22): given the narrow study aim, focused interview guide, dense participant accounts, and recruitment strategy emphasizing variation, 10 interviews were considered to provide sufficient information power for thematic analysis. Trustworthiness was supported through prolonged engagement with each transcript, a documented audit trail (codebook, memos, joint display), and transparent description of reflexive positioning (23). Member checking was not performed; this is acknowledged as a limitation.

### Quantitative data analysis

Quantitative analyses were descriptive. Continuous variables are presented as mean (SD) with 95% confidence intervals where appropriate, and categorical variables as counts and percentages. Analyses were performed in Prism 9.5.1 (GraphPad Software, Boston, MA, USA). As the study was descriptive and exploratory, no inferential hypothesis testing against external reference datasets was prespecified.

### Integration

Integration followed the convergent design described above and combined merging at 2 levels (13, 14, 28). At the theme level, USER-P subscale and domain scores were considered alongside the thematic findings to identify convergence, divergence, and expansion, summarized in a joint display (see Table III). At the case level, the 10 interview participants – each of whom had also completed the USER-P – were assembled into a mixed-methods matrix (see Table IV) in which their individual USER-P profiles were juxtaposed with their dominant qualitative emphasis, and each case was coded for the relationship between the quantitative and qualitative data (convergence/confirmation, divergence/discordance, or expansion) relative to the cohort means. Patterns across cases were then examined to derive meta-inferences.

### Ethics

Under Danish legislation (the Act on Research Ethics Review of Health Research Projects), survey-based research without intervention or biological material does not require approval from a regional research ethics committee, and this study was therefore not submitted to a committee. Processing of personal data was registered with the North Denmark Region in accordance with applicable data protection procedures. All participants provided informed consent electronically for the survey and separately for the interviews. The study was conducted in accordance with the principles of the Declaration of Helsinki.

## RESULTS

### Participant characteristics

A total of 108 individuals completed the online survey (Table I). Seventy-nine respondents (73%) were women and 29 (27%) were men. Mean age was 51.6 (SD 14.1) years and mean disease duration was 11.6 (SD 12.8) years. Seventy-four respondents indicated willingness to be interviewed, from whom a purposive subsample of 10 participants was selected. The interview subsample comprised 7 women and 3 men with mean age 52.8 (SD 12.4) years and mean disease duration 12.1 (SD 9.6) years. Four interviews took place in participants’ homes, 3 in a local cafe, and 3 at the hospital. Participants represented a range of occupational situations, including reduced-hours office work, self-employment, manual work, project management, teaching, and retirement.

**Table I:** Characteristics of survey respondents and interview participants

Characteristic	Survey (*n*=108)	Interview subsample (*n*=10)
Women, *n* (%)	79 (73)	7 (70)
Men, *n* (%)	29 (27)	3 (30)
Age, years, mean (SD)	51.6 (14.1)	52.8 (12.4)
Disease duration, years, mean (SD)	11.6 (12.8)	12.1 (9.6)
Interview setting, *n*	–	Home 4; cafe 3; hospital 3
Occupational situations represented	Not collected as discrete categories	Reduced-hours office work; self-employment; manual work; project management; teaching; retirement

The survey sample included all participants completing the online survey. The interview subsample was selected purposively from survey respondents who indicated willingness to be interviewed.

SD: standard deviation.

### Participation outcomes

**Fig. 1:**
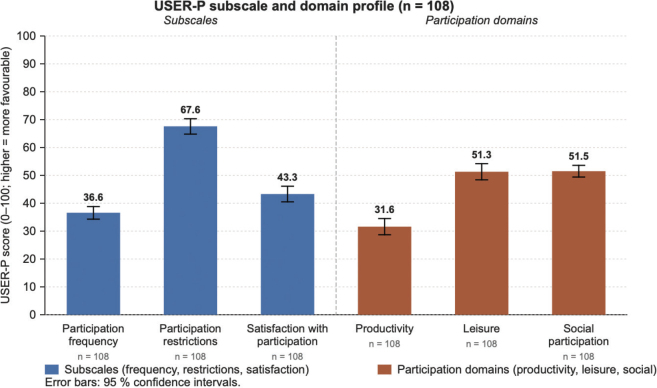
Utrecht Scale for Evaluation of Rehabilitation–Participation (USER-P) subscale and domain profile in adults with isolated cervical dystonia (*n*=108). Bar chart showing mean scores with 95% confidence intervals for participation frequency, participation restrictions, satisfaction with participation, and the productivity, leisure, and social domains. The y-axis ranges from 0 to 100; higher scores indicate more favourable participation.

USER-P outcomes are summarized in Table II and Fig. 1. Mean participation frequency was 36.6 (SD 11.6; 95% CI 34.3–38.8), indicating limited engagement across work, household, leisure, and social activities. Mean restrictions score was 67.6 (SD 14.3; 95% CI 64.8–70.3), indicating the presence of restrictions but a comparatively more favourable profile than the frequency and satisfaction subscales. Mean satisfaction score was 43.3 (SD 14.3; 95% CI 40.5–46.1), indicating low satisfaction with current participation. Across domains, the productivity score was markedly the lowest (mean 31.6, SD 14.8; 95% CI 28.7–34.5), whereas leisure (mean 51.3, SD 14.8; 95% CI 48.4–54.2) and social participation (mean 51.5, SD 10.8; 95% CI 49.4–53.6) were of comparable magnitude and indicated less pronounced but still constrained participation. Taken together, the USER-P profile indicates a broad participation burden, most marked in productivity, rather than restriction confined to a single area of everyday life.

**Table II:** Subscale and domain scores on the Utrecht Scale for Evaluation of Rehabilitation–Participation (USER-P) among respondents with isolated cervical dystonia (*n* = 108)

USER-P scale	Score range	Mean (SD)	95% CI
Subscale: Participation frequency	0–100	36.6 (11.6)	34.3–38.8
Subscale: Participation restrictions	0–100	67.6 (14.3)	64.8–70.3
Subscale: Satisfaction with participation	0–100	43.3 (14.3)	40.5–46.1
Domain: Productivity	0–100	31.6 (14.8)	28.7–34.5
Domain: Leisure	0–100	51.3 (14.8)	48.4–54.2
Domain: Social participation	0–100	51.5 (10.8)	49.4–53.6

Higher scores indicate more favourable participation outcomes on every scale.

SD: standard deviation; CI: confidence interval; USER-P: Utrecht Scale for Evaluation of Rehabilitation–Participation.

### Qualitative findings

Three interrelated themes were generated: (*i*) living with unpredictability; (*ii*) invisibility and contested recognition; and (*iii*) a symptom-focused system in a participation-focused life. Illustrative quotations are given below; participant identifiers (P01–P10) refer to interview participants.

*Living with unpredictability.* Participants described fluctuating pain, muscle contractions, postural deviation, and fatigue as central barriers to participation, with the treatment cycle, time of day, and accumulating workload structuring what was possible. Symptoms were not experienced as static; rather, variability undermined planning and confidence. Several participants described an internal calculation preceding everyday choices.

*It depends very much on where I am in the treatment cycle. When the injections are working, I can almost plan like other people. When they wear off, the neck starts pulling, the pain takes over, and I become careful about promising anything. (P01)*

*In the morning I may think I can teach, pick up the children, cook dinner, and answer emails. By the afternoon, pain and fatigue may make that impossible. (P06)*

Anticipatory withdrawal was a recurrent response to fluctuation: declining invitations early, narrowing role commitments, and avoiding situations whose course could not be predicted (P06, P10). Unpredictability therefore affected participation not only through bodily symptoms themselves, but also through uncertainty and pre-emptive constraint of valued activities.

*Invisibility and contested recognition.* Participants frequently described cervical dystonia as an under-recognised and partly invisible condition. Family members, employers, public authorities, and some healthcare professionals were reported to underestimate its impact because outward appearance did not reveal the underlying difficulty, particularly when symptoms were managed or temporarily suppressed. Concealment was both a coping strategy and a source of additional labour.

*Clothes, posture, and being busy can hide a lot. But they also make people assume I am not affected. (P02)*

*Some think it is stress. Some think it is a bad neck. A few make jokes about looking the wrong way. (P08)*

Recognition was particularly contested when capacity fluctuated. Performing competently on a good day made it harder for colleagues, employers, and assessment systems to credit the limitations encountered on bad days:

*Because I can function well some days, it is hard for them to understand why I need accommodations on others. The variability makes me look inconsistent. (P06)*

Concealment, fluctuation, and under-recognition together produced a sense of reduced legitimacy in occupational and social contexts and a reluctance to seek accommodations or support.

*A symptom-focused system in a participation-focused life.* Many participants described available healthcare and support systems as more readily addressing motor symptoms than wider participation problems. Botulinum toxin treatment was commonly described as helpful and was not questioned, but the surrounding system was experienced as narrow.

*Competent but narrow. The injections are technically good, and I appreciate that. But there is rarely a plan beyond the next injection. (P09)*

*It would make sense if injections and rehabilitation spoke to each other. (P10)*

Few participants reported access to coordinated rehabilitation, psychosocial support, vocational guidance, or systematic assessment of participation. Several articulated a preference for a structured participation-oriented conversation rather than additional clinical procedures: “Ask what I have stopped doing. That tells you more than asking only about pain from zero to ten” (P10). The gap between symptom-focused treatment and everyday support needs was experienced as frustrating, isolating, and inconsistent with the actual scope of the condition.

### Integration of quantitative and qualitative findings

Integrated findings are summarized in the joint display in Table III. The qualitative themes contextualized and partly explained the USER-P profile. The pattern of low participation frequency and low satisfaction was consistent with accounts of pre-emptive withdrawal from valued activities under conditions of fluctuating capacity. The markedly low productivity score was reflected in narratives of constrained work participation and reduced household role. Although the leisure and social participation domains scored higher than productivity, qualitative accounts of restricted hobbies and an experience of leisure as “logistics” (P07), alongside constrained, planned, and effortful social participation, indicated that these subscale scores did not reflect absence of difficulty; rather, ostensibly preserved participation was achieved at considerable cost. The moderate score on the restrictions subscale was illuminated by the qualitative finding that participation was limited not only by what participants could do, but also by whether everyday life could be organized, anticipated, and socially supported. Taken together, the integrated findings indicate that participation in cervical dystonia is shaped by an interaction between symptom burden, fluctuating capacity, social recognition, and the structure of available care, and that none of these dimensions is captured fully by symptom-focused assessment alone.

**Table III:** Joint display of quantitative and qualitative findings

USER-P finding	Related qualitative theme	Integrated interpretation
Low participation frequency (36.6) and low satisfaction (43.3) across daily activities	Living with unpredictability: fluctuating symptoms, treatment-cycle dependence, and anticipatory withdrawal	Reduced engagement and dissatisfaction reflect pre-emptive narrowing of activities under conditions of unpredictable capacity, not only lower momentary ability
Lowest domain score in productivity (31.6); leisure (51.3) and social participation (51.5) scored similarly and were less restricted	Invisibility and contested recognition: concealment, fluctuating visibility, and under-recognition by family, employers, healthcare, and authorities	The markedly low productivity score is consistent with constrained work and household participation amid limited social readability of the condition; the higher but still constrained leisure and social participation domains are consistent with effortful, hidden, and selectively disclosed engagement, where preserved subscale scores do not preclude meaningful restriction in valued roles
Leisure (51.3) and social participation (51.5) scored similarly and higher than productivity, but were not unrestricted	Constrained social participation managed through planning, seating choice, controlled exposure, and selective disclosure	Higher leisure and social subscale scores may be retained quantitatively while remaining effortful and reduced in spontaneity, indicating that a higher subscale score does not preclude meaningful participation restriction
Moderate restrictions subscale score (67.6) despite low frequency and satisfaction	A symptom-focused system in a participation-focused life: competent injection treatment but limited access to coordinated rehabilitation, vocational support, and participation-oriented assessment	Restriction is shaped not only by individual capacity but also by what care, accommodations, and support are available; available services partially mitigate restriction without addressing participation

Higher USER-P scores indicate more favourable outcomes. The display integrates findings at the level of interpretation.

USER-P: Utrecht Scale for Evaluation of Rehabilitation–Participation.

Case-level integration is presented in the mixed-methods matrix (Table IV). Two patterns recurred. In a convergent cluster (P05, P10), uniformly low USER-P profiles matched narratives of high symptom burden and relinquished valued roles. In a discordant cluster (P02, P06, P08, P09), relatively preserved participation frequency and productivity scores coexisted with low satisfaction and accounts of concealed effort, endurance, or formal workplace accommodation. Two participants with near-identical USER-P profiles (P02, a woman managing professional concealment, and P08, a man enduring manual work) described markedly different routes to closely similar scores. This juxtaposition indicated that the frequency and productivity subscales may under-detect restriction in higher-functioning or concealing participants, whereas the satisfaction subscale and the qualitative accounts captured it.

**Table IV:** Case-level mixed-methods matrix: individual USER-P profiles and qualitative emphasis for the 10 interview participants

Case	F/R/S	Prod/Leis/Soc	Dominant qualitative emphasis	Quantitative-qualitative relation
P01 (Linda)	36.4/66.7/48.0	23.5/60.0/51.1	Work preserved through invisible environmental adaptation; end-of-cycle worsening	Convergent-expansion: low productivity matches reduced-hours work; preserved leisure/social sustained by hidden adaptation not visible in scores
P02 (Carol)	47.3/66.7/44.0	47.1/50.0/53.3	Professional concealment; high self-monitoring; running as contested freedom	Discordant: relatively favourable frequency/productivity coexist with effortful concealment and only average satisfaction; scores overstate ease
P03 (Janet)	29.1/72.7/46.0	20.6/46.7/62.2	Diagnostic delay (mistaken for Parkinson’s disease); maintained but planned social life	Expansion: high social score qualified by effortful maintenance and reluctance to burden family; low productivity reflects retirement
P04 (Karen)	29.1/72.7/46.0	17.6/56.7/46.7	Explanatory models; public self-consciousness; stigma; peer-support ambivalence	Convergent-expansion: below-average social consistent with self-surveillance; stigma as anticipatory self-monitoring not captured by scores; low productivity reflects retirement
P05 (Patricia)	25.5/63.6/40.0	14.7/46.7/48.9	Pain-dominant; early retirement after cancer; self-management; contested agency	Convergent: uniformly low, unfavourable profile matches high symptom and comorbidity burden
P06 (Anne)	41.8/69.7/24.0	38.2/38.3/48.9	Teacher and parent; role accumulation; fatigue; anticipatory withdrawal	Discordant: above-average productivity/frequency but markedly low satisfaction and sacrificed leisure; restriction concentrated in satisfaction and leisure, not activity counts
P07 (Eva)	32.7/60.6/28.0	38.2/35.0/40.0	Home-based self-employment; isolation; social withdrawal; online support	Convergent-expansion: low leisure/social and satisfaction match withdrawal; home-based work preserves productivity at the cost of visibility and spontaneity
P08 (Niels)	47.3/66.7/44.0	41.2/53.3/53.3	Manual work; masculinity; occupational safety; reluctance to request accommodation	Discordant: favourable scores sustained by endurance and non-disclosure; gendered reluctance masks unmet need (near-identical scores to P02, different route)
P09 (Thomas)	40.0/78.8/40.0	50.0/50.0/46.7	IT project manager; formal accommodations; cognitive load; support-system gap	Discordant: highest productivity and lowest restriction reflect successful accommodation, not absence of need; cognitive load and unmet coordination invisible to scores
P10 (Mikkel)	25.5/60.6/32.0	20.6/35.0/51.1	Lost valued leisure and identity roles (driving, music); stopped activities	Convergent-expansion: low leisure and satisfaction match relinquished valued roles; identity loss and stopped activities add a dimension beyond scores

Higher scores indicate more favourable outcomes on every scale. Cohort means (*n*=108): F 36.6, R 67.6, S 43.3, Prod 31.6, Leis 51.3, Soc 51.5. Relation classifies each case as convergent (quantitative and qualitative data agree), discordant (they conflict), or expansion (qualitative data reveal a dimension not visible in the scores), judged relative to cohort means.

USER-P: Utrecht Scale for Evaluation of Rehabilitation-Participation; F: participation frequency; R: participation restrictions; S: satisfaction with participation; Prod: productivity; Leis: leisure; Soc: social participation.

## DISCUSSION

This mixed-methods study found that adults with isolated cervical dystonia reported restricted participation across multiple domains of daily life. The USER-P profile was characterized by low participation frequency, moderate perceived restrictions, and low satisfaction with participation; productivity was the most affected domain, while leisure and social participation scored similarly and indicated less pronounced but still constrained engagement. Qualitative findings indicated that these participation problems were experienced in relation to symptom unpredictability, invisibility and contested recognition, and a care system experienced as symptom-focused rather than participation-focused.

Beyond describing how the integration was performed, the contribution of the integration itself to knowledge generation warrants explicit discussion. Three insights would not have emerged from either component alone. First, where the data converged – uniformly low USER-P profiles alongside narratives of high symptom burden and relinquished roles – each component corroborated the other, strengthening confidence that low scores reflected genuine restriction rather than response style. Second, the discordant cases were analytically the most productive: relatively preserved frequency and productivity scores coexisting with low satisfaction and accounts of concealed effort indicate that these subscales register whether activities occur, not what they cost. The survey alone would have suggested moderately preserved engagement in these participants, while the interviews alone could not have shown that satisfaction, rather than frequency, was the subscale departing most clearly from other diagnostic groups. Third, the qualitative component expanded the quantitative picture by locating part of the restriction outside the individual – in social recognition and the organization of care – dimensions that a self-report instrument referenced to individual activity cannot capture by design. The resulting meta-inference is that participation in cervical dystonia is better characterized as high-effort, low-reward engagement than as low participation per se; this reframing emerged only from the combination of the 2 datasets.

These results extend previous literature on non-motor symptoms and quality of life in cervical dystonia (3–7) by shifting the analytical lens from symptoms and generic quality of life to participation as defined by the ICF (9). Recent qualitative studies in cervical and neck dystonia describe active but challenging lives shaped by pain, fatigue, stress, altered self-image, stigma, isolation, and psychological distress (10, 11), and the present qualitative themes are broadly consistent with that emerging picture. The specific contribution of the present work is twofold. First, it provides a participation-specific quantitative description using a validated participation instrument. Second, it integrates that quantitative description with qualitative accounts through an explicit joint display, so that the USER-P profile is interpreted in light of how participation is produced, constrained, and negotiated in daily life rather than as a stand-alone metric.

To place the present findings in cross-diagnostic context, the USER-P profile observed in this sample can be compared with diagnostic groups in published USER-P cohorts. In secondary analyses of 8 Dutch USER-P studies by Mol et al., reported subscale means were lowest in progressive neurological and neuromuscular diseases (Restrictions 52.2; Satisfaction 56.3) and highest in acute coronary syndrome (94.6; 74.6), with stroke (77.5; 66.7), spinal cord injury (76.4; 68.8), other acquired brain injury (81.1; 68.0), and subarachnoid haemorrhage (68.5; 65.5) intermediate (24). Single-condition cohorts have reported comparable scores in Norwegian spinal cord injury (Participation Frequency 30.7; Restrictions 70.3) (25), spinal muscular atrophy (Restrictions 61.9; Satisfaction 72.5) (26), and subarachnoid haemorrhage at 3 months (Restrictions 68.6) (27). Within this landscape, the cervical dystonia profile observed here is distinctive on 3 points. Participation Frequency (36.6) sat at the high end of the cross-diagnostic range, comparable to acute coronary syndrome (36.3). Restrictions (67.6) approached the subarachnoid haemorrhage range and were more pronounced than in stroke, acquired brain injury, or spinal cord injury; only progressive neurological disease scored worse. Satisfaction (43.3) was substantially lower than the lowest diagnostic group reported by Mol et al. (progressive neurological disease, 56.3), and lower than any single-condition USER-P cohort identified above. This pattern – preserved frequency alongside markedly low satisfaction and pronounced perceived restriction – corresponds to the integrated meta-inference of high-effort, low-reward engagement developed above. The cervical dystonia profile therefore does not coincide with any single condition in the published USER-P literature, supporting participation-specific outcome assessment rather than reliance on symptom-focused measures alone. Direct comparison of the satisfaction-based Productivity domain score (31.6) with the Productivity scores of Mol et al. is limited because their domain scores aggregated items across all 3 subscales, whereas the present domain scores are derived from satisfaction items only.

The integrated findings also help explain why participation may remain restricted despite access to symptomatic treatment. Botulinum toxin is effective for many patients and remains first-line treatment (2), but symptom reduction alone may not resolve participation problems if individuals continue to face uncertainty, stigma, or inadequate accommodation. This interpretation is consistent with prior observations that emotional well-being, pain, fatigue, and related non-motor factors are important contributors to disability in cervical dystonia (5–7), and with recent rehabilitation reviews arguing that management often needs to extend beyond injection treatment towards individualized, multidisciplinary rehabilitation delivered as an adjunct to standard care (8).

From a rehabilitation perspective, the results are consistent with a broader clinical assessment strategy, in which patient-reported participation could be considered alongside impairment-focused examination. Whether such an assessment would translate into improved outcomes was not tested here. The findings also point towards the potential value of interdisciplinary rehabilitation including education, self-management support, psychosocial assessment, vocational support, and interventions that acknowledge fluctuating or non-visible disability (8). These implications are descriptive rather than prescriptive: the present study was not designed to test rehabilitation interventions, and the directionality of any causal relationship between participation and care structure cannot be inferred from cross-sectional data.

The study has several strengths. It used a participation-specific outcome measure and combined it with qualitative interviews, so that quantitative scores could be interpreted in the light of lived experience. The mixed-methods design was appropriate to the research question, and integration was made explicit through a joint display. The qualitative component followed an established reflexive thematic analysis framework with a documented audit trail and reflexive memos. Integration was achieved at both theme and case level, including a mixed-methods matrix linking each interviewee’s USER-P profile to their account, which allowed convergence and discordance to be examined analytically rather than only interpretively.

Several limitations should be acknowledged. First, the sample was recruited through patient association channels, social media, and clinical networks, which may have favoured participation by individuals who were more symptomatic, more health-engaged, or more motivated to describe difficulties; generalizability is therefore uncertain, and selection bias cannot be excluded. Second, diagnosis and clinical characteristics were self-reported, and no independent verification against current consensus criteria for isolated cervical dystonia (17), or clinical rating of dystonia severity, pain, or psychiatric symptoms, was available. Third, the quantitative component was descriptive, so the study cannot establish determinants of participation or support causal interpretations. Fourth, the qualitative component was conducted by a single analyst. Reflexive thematic analysis acknowledges single-analyst designs as methodologically valid when reflexivity and an audit trail are maintained (20, 21), and information power was used as a sample-size rationale (22); nevertheless, additional analysts could have broadened the interpretive lens, and member checking was not performed. Finally, additional potentially relevant variables, including employment status, treatment timing, comorbidity, and socioeconomic position, were not analysed. In addition, the case-level integration was *post hoc*: although the interview subsample was purposively selected using survey-derived characteristics, selection was not stratified by USER-P scores, and the mixed-methods matrix was constructed after data collection. The convergent and discordant patterns identified in the matrix should therefore be regarded as hypothesis-generating rather than confirmatory – the coding of convergence and discordance was performed by a single analyst and is open to confirmation bias, and with 10 cases apparent clusters may reflect chance. Prospective designs that stratify qualitative sampling by questionnaire scores could test these patterns directly.

In conclusion, adults with isolated cervical dystonia in this sample reported low participation frequency, moderate perceived restrictions, and low satisfaction with participation across work, leisure, and social life. These participation problems were not captured adequately by a symptom-focused perspective alone, or fully by questionnaire scores, but were experienced in relation to fluctuating symptoms, invisibility, and contested recognition, and a care system organized primarily around symptoms. Patient-reported participation may therefore be a useful complementary outcome domain in both clinical assessment and rehabilitation-oriented research on cervical dystonia.

## Supplementary Material



## References

[1] Albanese A, Bhatia K, Bressman SB, Delong MR, Fahn S, Fung VSC, et al. Phenomenology and classification of dystonia: a consensus update. Mov Disord 2013; 28: 863–873. 10.1002/mds.25475 23649720 PMC3729880

[2] Castelão M, Marques RE, Duarte GS, Rodrigues FB, Ferreira J, Sampaio C, et al. Botulinum toxin type A therapy for cervical dystonia. Cochrane Database Syst Rev 2017; 12: CD003633. 10.1002/14651858.CD003633.pub3 29230798 PMC6486222

[3] Ray S, Pal PK, Yadav R. Non-motor symptoms in cervical dystonia: a review. Ann Indian Acad Neurol 2020; 23: 449–457. 10.4103/aian.AIAN_27_20 33223660 PMC7657286

[4] Girach A, Vinagre Aragon A, Zis P. Quality of life in idiopathic dystonia: a systematic review. J Neurol 2019; 266: 2897–2906. 10.1007/s00415-018-9119-x 30460447 PMC6851210

[5] Klingelhoefer L, Kaiser M, Sauerbier A, Untucht R, Wienecke M, Mammadova K, et al. Emotional well-being and pain could be a greater determinant of quality of life compared to motor severity in cervical dystonia. J Neural Transm (Vienna) 2021; 128: 305–314. 10.1007/s00702-020-02274-z 33146753 PMC7969693

[6] Han V, Skorvanek M, Smit M, Turcanova Koprusakova M, Hoekstra T, van Dijk JP, et al. Prevalence of non-motor symptoms and their association with quality of life in cervical dystonia. Acta Neurol Scand 2020; 142: 613–622. 10.1111/ane.13304 32579704

[7] Zetterberg L, Lindmark B, Söderlund A, Åsenlöf P. Self-perceived non-motor aspects of cervical dystonia and their association with disability. J Rehabil Med 2012; 44: 950–954. 10.2340/16501977-1055 23069793

[8] Kamo H, Nagaki K, Kraus AR, Warren L, Wagle Shukla A. Neurorehabilitation in dystonia care: key questions of who benefits, what modalities, and when to intervene. Dystonia 2025; 4: 14695. 10.3389/dyst.2025.14695 40855851 PMC12376834

[9] World Health Organization. International Classification of Functioning, Disability and Health: ICF. Geneva: World Health Organization; 2001.

[10] Zetterberg L, Niemi Andersson E, Åsenlöf P, Nyholm D, de Roos P, Bring A. “I’m still the person I am. Not the body it has become.” An active but challenging life with cervical dystonia. Physiother Theory Pract 2025; 41: 763–771. 10.1080/09593985.2024.2359495 38814175

[11] McCormack D, O’Keeffe F, Eccles FJR. Lived experiences of psychological distress in neck dystonia. Disabil Rehabil 2025; 47: 5816–5828. 10.1080/09638288.2025.2536719 40728912

[12] Post MWM, van der Zee CH, Hennink J, Schafrat CG, Visser-Meily JMA, van Berlekom SB. Validity of the Utrecht Scale for Evaluation of Rehabilitation-Participation. Disabil Rehabil 2012; 34: 478–485. 10.3109/09638288.2011.608148 21978031

[13] O’Cathain A, Murphy E, Nicholl J. The quality of mixed methods studies in health services research. J Health Serv Res Policy 2008; 13: 92–98. 10.1258/jhsrp.2007.007074 18416914

[14] Tovin MM, Wormley ME. Systematic development of standards for mixed methods reporting in rehabilitation health sciences research. Phys Ther 2023; 103: pzad084. 10.1093/ptj/pzad084 37672215

[15] von Elm E, Altman DG, Egger M, Pocock SJ, Gøtzsche PC, Vandenbroucke JP. The Strengthening the Reporting of Observational Studies in Epidemiology (STROBE) statement: guidelines for reporting observational studies. Lancet 2007; 370: 1453–1457. 10.1016/S0140-6736(07)61602-X 18064739

[16] Tong A, Sainsbury P, Craig J. Consolidated criteria for reporting qualitative research (COREQ): a 32-item checklist for interviews and focus groups. Int J Qual Health Care 2007; 19: 349–357. 10.1093/intqhc/mzm042 17872937

[17] Albanese A, Bhatia KP, Cardoso F, Comella C, Defazio G, Fung VSC, et al. Isolated cervical dystonia: diagnosis and classification. Mov Disord 2023; 38: 1367–1378. 10.1002/mds.29387 36989390 PMC10528915

[18] Harris PA, Taylor R, Thielke R, Payne J, Gonzalez N, Conde JG. Research electronic data capture (REDCap): a metadata-driven methodology and workflow process for providing translational research informatics support. J Biomed Inform 2009; 42: 377–381. 10.1016/j.jbi.2008.08.010 18929686 PMC2700030

[19] Harris PA, Taylor R, Minor BL, Elliott V, Fernandez M, O’Neal L, et al. The REDCap consortium: building an international community of software platform partners. J Biomed Inform 2019; 95: 103208. 10.1016/j.jbi.2019.103208 31078660 PMC7254481

[20] Braun V, Clarke V. Using thematic analysis in psychology. Qual Res Psychol 2006; 3: 77–101. 10.1191/1478088706qp063oa

[21] Braun V, Clarke V. One size fits all? What counts as quality practice in (reflexive) thematic analysis? Qual Res Psychol 2021; 18: 328–352. 10.1080/14780887.2020.1769238

[22] Malterud K, Siersma VD, Guassora AD. Sample size in qualitative interview studies: guided by information power. Qual Health Res 2016; 26: 1753–1760. 10.1177/1049732315617444 26613970

[23] Nowell LS, Norris JM, White DE, Moules NJ. Thematic analysis: striving to meet the trustworthiness criteria. Int J Qual Methods 2017; 16: 1609406917733847. 10.1177/1609406917733847

[24] Mol TI, van Bennekom CA, Schepers VP, Ter Hoeve N, Kruitwagen-van Reenen ET, Visser-Meily JM, et al. Differences in societal participation across diagnostic groups: secondary analyses of eight studies using the Utrecht Scale for Evaluation of Rehabilitation-Participation. Arch Phys Med Rehabil 2021; 102: 1735–1745. 10.1016/j.apmr.2021.02.024 33757804

[25] Halvorsen A, Pape K, Post MWM, Biering-Sørensen F, Mikalsen S, Hansen AN, et al. Participation and quality of life in persons living with spinal cord injury in Norway. J Rehabil Med 2021; 53: jrm00217. 10.2340/16501977-2858 34232321 PMC8638721

[26] Kruitwagen-van Reenen ET, van der Pol L, Schröder C, Wadman RI, van den Berg LH, Visser-Meily JMA, et al. Social participation of adult patients with spinal muscular atrophy: frequency, restrictions, satisfaction, and correlates. Muscle Nerve 2018; 58: 805–811. 10.1002/mus.26201 30028531

[27] Kruisheer EM, Huenges Wajer IMC, Visser-Meily JMA, Post MWM. Course of participation after subarachnoid hemorrhage. J Stroke Cerebrovasc Dis 2017; 26: 1000–1006. 10.1016/j.jstrokecerebrovasdis.2016.11.124 28109733

[28] Fetters MD, Curry LA, Creswell JW. Achieving integration in mixed methods designs: principles and practices. Health Serv Res 2013; 48: 2134–2156. 10.1111/1475-6773.12117 24279835 PMC4097839

