# Supplementary material for: PARTICIPATION RESTRICTIONS IN ISOLATED CERVICAL DYSTONIA: A CONVERGENT MIXED-METHODS STUDY
Source: J Rehabil Med. 2026 Jul 9;58:46205. doi: 10.2340/jrm.v58.46205 (PMC13358788; doi:10.2340/jrm.v58.46205)
Supplement: Supplementary file 1 [file JRM-58-46205-s1.pdf]

Appendix S1. Interview guide

Participation restrictions in isolated cervical dystonia: a convergent mixed-methods study

The following semi-structured interview guide was used in face-to-face interviews with adults with isolated cervical dystonia. It was developed to support open exploration of participation in everyday life rather than to elicit predetermined responses. The author used the guide flexibly, following the participant's own framing where this opened relevant material, and probing for concrete examples and contextual detail where useful. Probes included: "Can you give me an example?", "How did that play out in everyday life?", "What did you do then?", and "What would have helped?".

Opening

Thank you for agreeing to share your experience. The aim of this conversation is to understand what it is like to live with cervical dystonia in everyday life — at work, at home, in leisure activities, and with other people. There are no right or wrong answers. We can stop or skip any question at any time.

Core questions

1. Can you describe when you first noticed symptoms and how you understood them at the time?
2. What are the most troublesome symptoms in everyday life?
3. Do symptoms fluctuate over the day or across the treatment cycle?
4. How does cervical dystonia affect work, household tasks, mobility, leisure, and social life?
5. How do other people respond to the condition, and what do they understand or misunderstand?
6. What has your contact with healthcare professionals and public support systems been like?
7. How do you manage symptoms yourself, and what forms of support have been useful?
8. What would better care or rehabilitation look like from your perspective?

Closing

Is there anything we have not talked about that you would like to add? Is there anything you would want clinicians and researchers to understand about cervical dystonia that we have not already covered?

Appendix S2. Participant matrix, working codebook, and condensed transcripts with analytic memos

Participation restrictions in isolated cervical dystonia: a convergent mixed-methods study

This supplementary file documents the qualitative audit trail for the interview component of the study. It contains: (i) a participant matrix summarising the ten interview participants; (ii) the working codebook used in the reflexive thematic analysis, with candidate codes grouped under the three themes generated through analysis; and (iii) condensed transcripts of each interview together with the analytic memo and candidate codes assigned to that participant. Names are pseudonyms; minor non-substantive edits were made to support readability of the condensed form, without altering meaning.

1. Participant matrix

The matrix summarises participants' pseudonym, sex (F = female; M = male), age, estimated disease duration, primary occupational situation at interview, and interview setting. Purposive sampling sought variation in age, sex, occupational situation, and self-reported symptom burden.

ID	Pseudonym	Sex / age (years)	Disease duration (years)	Occupational situation	Interview setting
**P01**	Linda	F / 55	9	Office assistant (public administration), reduced hours	Home
**P02**	Carol	F / 53	7	Co-owner, small furniture business	Café
**P03**	Janet	F / 71	15	Retired office worker	Hospital
**P04**	Karen	F / 70	8	Retired health visitor	Home
**P05**	Patricia	F / 51	2	Early retirement following breast	Hospital

				cancer treatment	
**P06**	Anne	F / 34	16	Schoolteacher; parent of young children	Home
**P07**	Eva	F / 42	13	Self-employed graphic designer; works from home	Café
**P08**	Niels	M / 47	18	Skilled manual worker	Café
**P09**	Thomas	M / 49	21	IT project manager	Hospital
**P10**	Mikkel	M / 56	12	Former driver and amateur musician	Home

2. Working codebook

Codes are presented as developed during analysis. The codebook was iteratively refined: initial inductive codes were generated during familiarisation and line-level reading, clustered into candidate themes, and reviewed against the dataset as a whole. Codes are grouped under the three themes generated through analysis. A small number of cross-cutting descriptive codes that indexed participation domains and adaptive strategies are listed separately at the end. The codebook is presented as it was used; codes are not mutually exclusive, and several participants contributed to multiple codes and themes.

Theme 1 — Living with unpredictability

Codes capturing fluctuation of symptoms over the treatment cycle and the day, anticipatory adjustment of behaviour, and the experiential and bodily texture of unpredictable capacity.

symptom unpredictability. Reports that symptoms (pain, pulling, posture, tremor) varied within and across days without reliable cues.

fluctuation. Day-to-day or week-to-week variation in capacity, distinct from background severity.

treatment cycle. Behaviour, planning, and mood organised around proximity to the next botulinum toxin injection.

anticipatory withdrawal. Pre-emptive declining of activities or commitments because of uncertain capacity.

fatigue. Energy depletion arising from symptom management or activity, distinct from sleepiness.

pain. Pain reported as a dominant or controlling symptom for everyday function.

planning. Effortful structuring of the day, seating, route, and breaks to make activity feasible.

cognitive load. Mental effort of monitoring head, pain, posture, and pacing alongside the actual task.

low satisfaction. Dissatisfaction with current level or quality of participation, even where activity is retained.

Theme 2 — Invisibility and contested recognition

Codes capturing the visibility and social readability of cervical dystonia, the labour of concealment, and the contested credibility of fluctuating disability in social and institutional contexts.

underrecognition. Family members, employers, authorities, or clinicians underestimating the condition's impact.

concealment. Active strategies to hide symptoms (clothing, posture, busy-ness, avoidance of disclosure).

self-consciousness. Awareness of one's own head position or appearance during interaction.

public self-consciousness. Concern about being observed in public settings (cinema, transport, stage).

professional identity. Performance of competence in professional settings, sometimes at the cost of recognition.

stigma. Anticipation of, or experience of, negative social evaluation linked to the condition.

masculinity. Gendered reluctance to request accommodation or admit limitation in occupational contexts.

occupational safety. Safety concerns where head posture or pain affects manual or operational work.

formal support systems. Encounters with workplace, insurance, or disability assessment systems that demand stable, binary descriptions of capacity.

work accommodation. Negotiated or unilateral changes to working pattern, environment, or tasks.

social withdrawal. Reduced spontaneous social participation, repeated cancellations, narrowing networks.

diagnostic delay. Time and uncertainty between symptom onset and a confirmed diagnosis.

Theme 3 — A symptom-focused system in a participation-focused life

Codes capturing how care, support, and informal resources were experienced as competent in symptomatic treatment but narrow in addressing participation, identity, and everyday life.

symptom-focused care. Clinical encounters experienced as concentrating on injection technique, dose, and side effects.

support-system gap. Absence of coordinated, participation-oriented assessment or rehabilitation.

coordinated rehabilitation. Desire or experience of integrated input from medicine, physiotherapy, vocational, and psychological domains.

physiotherapy. Use of physiotherapy for posture, pacing, or positioning, often self-arranged.

complementary therapy. Use of acupuncture, massage, laser, heat, or similar adjuncts.

self-management. Daily strategies for symptom mitigation and activity pacing.

peer support. Helpful contact with others living with chronic illness, including non-dystonia peer groups.

peer support ambivalence. Mixed appraisal of dystonia-specific peer contact, e.g. fear of seeing worse outcomes.

online support. Use of online communities for information and recognition not available offline.

family support. Practical and emotional support from partners or relatives, and its limits.

explanatory model. Participants' own causal accounts of how the dystonia arose.

paternalistic concern. Well-intentioned advice to conserve energy or limit activity that reduced agency.

agency. Active claiming of roles, identities, or choices despite the condition.

long-term adaptation. Cumulative reorganisation of work, identity, and routines over many years.

Cross-cutting descriptive codes (participation domains and adaptations)

Codes used descriptively to index domains of participation and concrete adaptive strategies, often co-occurring with theme codes above.

work participation. Engagement in paid work, including reduced hours, role changes, and self-employment.

home-based work. Work conducted from home as accommodation and as a source of isolation.

flexible work. Formal flexibility arrangements (hours, location, leave).

manual work. Physically demanding occupational settings with limited postural flexibility.

parenting. Demands and gratifications of parenting with a fluctuating condition.

leisure restriction. Reduced engagement in leisure activities and loss of valued hobbies.

leisure identity. Leisure activities understood as constitutive of identity, not merely recreation.

social participation. Engagement in social roles, gatherings, and relationships.

driving. Driving as both an enabling adaptation (headrest support) and a source of fatigue on long trips.

environmental adaptation. Modification of chairs, lighting, seating, and positioning to support activity.

physical activity. Use of running, walking, Pilates, or comparable activity as health behaviour and identity.

participation outcome. Stopping or substantially changing an activity as a clinically meaningful outcome.

3. Condensed transcripts with analytic memos

For each interview participant, the section presents: (a) participant profile; (b) interview setting; (c) dominant analytic emphasis; (d) the condensed transcript; (e) the analytic memo; and (f) the candidate codes assigned. The condensed transcript preserves the structure and meaning of the conversation while removing repetition, filler, and identifying detail. Speaker labels are "I" for interviewer (SBE) and the participant pseudonym for the interviewee.

P01. Linda

Profile. Woman, 55 years; disease duration estimated at 9 years; lives alone; reduced-hours office assistant in public administration. She uses a height-adjustable desk and an office chair with neck support; driving may relieve symptoms through head support.
Interview setting. Interview at home on an ordinary weekday.
Dominant analytic emphasis. Work participation, environmental adaptation, end-of-cycle worsening, and frustration with injection intervals.

Condensed transcript

I: Can you begin by describing what cervical dystonia changes in an ordinary week?
Linda: It depends very much on where I am in the treatment cycle. When the injections are working, I can almost plan like other people. When they wear off, the neck starts pulling, the pain takes over, and I become careful about promising anything.
I: What happens at work when that begins?
Linda: I work reduced hours already, and I have arranged my office around the neck. The chair has support, the desk can move up and down, and I try not to sit in one position too long. But if the neck acts up, I may need to call in sick. That is difficult, because the work itself is not the problem. The unpredictability is.
I: Do your colleagues understand why you have to arrange things like that?
Linda: Some do, but it is not easy to explain. People can see me sitting there and typing, and then they think I am fine. They do not see the calculation behind it - the chair, the head support, the breaks, the way I avoid meetings late in the day.
I: You mentioned driving can sometimes help.

Linda: Yes, because I can press the head against the headrest. It is strange, because driving should be demanding, but that support gives me some control. It is also why I notice so much when a chair is bad. The environment decides more than people think.
I: How do you experience waiting for treatment?
Linda: That is one of the hardest things. Twelve weeks is long, and if the appointment is delayed, I start arranging life around the symptoms. I know the injections cannot be magical, but the interval matters. When it becomes more than twelve weeks, I feel that my everyday life is being stretched beyond what I can manage.
I: What would make care feel more supportive?
Linda: Someone asking about my work and my daily life, not only whether the dose worked. I would like the system to understand that the injection is connected to whether I can keep my job, see people, and live alone without everything becoming a project.

Analytic memo

- Linda illustrates participation as a function of symptom timing and environmental control rather than impairment alone.
- The work role is partially preserved, but only through adaptations that remain invisible to others.
- Her account supports the manuscript interpretation that participation is limited by whether everyday life can be organised and anticipated.

Candidate codes

symptom unpredictability, treatment cycle, work participation, environmental adaptation, underrecognition, support-system gap

P02. Carol

Profile. Woman, 53 years; disease duration estimated at 7 years; co-owner of a small furniture business; adult children; maintains a professional appearance and continues running.
Interview setting. Cafe interview during lunch break.
Dominant analytic emphasis. Professional identity, concealment, social exposure, physical activity, and competing explanations of illness.

Condensed transcript

I: You have kept a very active working life. How does dystonia fit into that?

Carol: It fits because I make it fit. I plan meetings, I travel when I must, and I keep the business going. But there is always a layer of monitoring myself. Is my head turning? Am I looking tense? Will the customer notice? It takes energy.
I: Does looking professional help?
Carol: Yes and no. Clothes, posture, and being busy can hide a lot. But they also make people assume I am not affected. If I arrive in a blazer and heels, people do not imagine that I have pain or that I have been thinking about my neck all morning.
I: How do you handle questions from customers or acquaintances?
Carol: Usually I do not invite them. I do not want the conversation to become about my neck. If people stare, I prefer to move on. It should take up as little attention as possible.
I: You still run. What does running mean to you?
Carol: It is freedom. It is one of the few situations where I feel the body is doing something normal, something I choose. But I also know that if I overdo it, pain and fatigue can follow. So even freedom has to be measured.
I: Have you developed an explanation for why the condition started?
Carol: Not really for myself. But I remember speaking to a younger woman with dystonia who thought it came from carrying heavy plates in the same position when she worked in Japan. I understand why people look for those explanations. You want the body to make sense.
I: What do you need from healthcare?
Carol: Less routine and more conversation. I understand the clinic is busy, and injections are important. But I also need to discuss how to live with it. What should I expect long term? How do I stay active without making things worse? Those questions do not fit easily into a fast appointment.

Analytic memo

- Carol demonstrates how preserved productivity can coexist with low satisfaction and high self-monitoring.
- Professional presentation becomes both a resource and a barrier to recognition.
- Her case links agency to concealment: she remains active, but participation is effortful and socially managed.

Candidate codes

professional identity, concealment, self-consciousness, physical activity, underrecognition, symptom-focused care

P03. Janet

Profile. Woman, 71 years; disease duration estimated at 15 years; retired office worker; married; active family and social life; initially treated as possible Parkinson disease because of head tremor and family history.

Interview setting. Hospital meeting room after several rescheduled appointments.

Dominant analytic emphasis. Diagnostic uncertainty, family support, maintained social participation, and the ambiguity of being outwardly functional.

Condensed transcript

I: When you think back to the beginning, how did you understand the symptoms?

Janet: It started with tremor and pain, and because my father had Parkinson disease, that was what I feared. My general practitioner also thought along those lines. I was treated as if it might be Parkinson disease before anyone named it dystonia.

I: What did the diagnosis change?

Janet: It gave a name to something that had felt vague and worrying. But a name is not the same as an explanation. I still had to understand what it meant for daily life.

I: How does it affect everyday activities now?

Janet: I am retired, so I do not have the pressure of work. But I am busy with family and friends. I still drive, and my husband and I manage. The problem is not always whether I can do something, but whether I can do it without planning around pain and fatigue.

I: Do people around you notice?

Janet: Family notices, but they also know me as the person who keeps going. Sometimes that makes it harder to say that I am tired or need to leave early. If you usually manage, people can forget that managing costs something.

I: What about social gatherings?

Janet: I enjoy them, but I choose where to sit, and I prefer not to be placed where I have to turn my head all evening. Small things like that matter. If I say no to something, people may think it is age, but often it is the neck.

I: How has treatment been for you?

Janet: The injections help. I am grateful for that. But the appointment can become very technical: where to inject, how much, side effects. I would not mind being asked more directly how it affects the rest of life.

Analytic memo

• Janet shows that social participation may be maintained while still constrained by planning, seating, fatigue, and reluctance to burden family.
• Her diagnostic story illustrates underrecognition even within healthcare when symptoms are interpreted through more familiar neurological categories.
• The case helps explain why a relatively higher social participation score does not imply absence of restriction.

Candidate codes

diagnostic delay, family support, social participation, planning, symptom-focused care, underrecognition

P04. Karen

Profile. Woman, 70 years; disease duration estimated at 8 years; retired health visitor; lives with husband near the coast; interested in sustainable living; uses positioning strategies in activities such as Pilates.
Interview setting. Home interview.
Dominant analytic emphasis. Explanatory models, public self-consciousness, complementary treatment, stigma, and ambivalence about peer support.

Condensed transcript

I: You have thought a lot about why the dystonia began. How do you understand it?
Karen: It came during a period when I was depressed and had stopped working. I was involved in a sustainable settlement group, and there was a conflict. I felt awful afterwards. Then I watched television in the same position for long periods, always over one shoulder. I think I provoked it because it became too much.
I: Does that explanation affect how you manage symptoms now?
Karen: Yes. I have always thought it needed to be unlearned. I place myself so I must look over the other shoulder. In Pilates I choose a specific place in the room. It is not only exercise; it is also arranging the body and the space.
I: How is it to be in public places?
Karen: Usually I manage. But in a crowded cinema recently, it became impossible not to think about it. If no one sits behind me, I forget it. When people are behind me, I become aware of my head and whether it disturbs them. Then the film is no longer the film.
I: Do you tell people about the condition?

Karen: Sometimes, but not always. There is no reason for people to speculate if they cannot see anything. I am glad when it is hidden, because then it is mine to carry.

I: You have also tried acupuncture.

Karen: Yes, and I did not tell everyone. Some people become uneasy about that. Even in my own family, it can provoke reactions. That is interesting, because it is not only the illness that is judged, but also the way you try to manage it.

I: What do you think about patient groups?

Karen: Support groups can be very good, because you hear other experiences. But I am also afraid of seeing people who are much worse. It can be empowering, but it can also be frightening if you start imagining that this is how it ends.

Analytic memo

- Karen is the clearest example of narrative meaning-making and an explanatory model that is personally coherent but not necessarily biomedical.
- Her public-cinema account shows stigma as anticipatory self-surveillance rather than only overt discrimination.
- Her ambivalence about support groups prevents an overly simple conclusion that peer contact is always empowering.

Candidate codes

explanatory model, environmental adaptation, public self-consciousness, stigma, complementary therapy, peer support ambivalence

P05. Patricia

Profile. Woman, 51 years; disease duration estimated at 2 years; early retirement after breast cancer treatment; uses heat, massage, exercise, and previously laser treatment for pain; participates in a cancer-related walking group.

Interview setting. Hospital office.

Dominant analytic emphasis. Pain, self-management, causal uncertainty, peer support, and contested agency.

Condensed transcript

I: What has been most difficult about living with cervical dystonia?

Patricia: Pain. The pulling is there, but pain is what controls the day. I have tried acupuncture, massage, laser treatment, exercises, heat - everything. When I am in a lot of pain, the heating pad is almost impossible to do without.
I: How do injections fit into that?
Patricia: They help, and I am not against them. But they do not remove everything. Between injections I still need strategies. Massage with a friend who is trained, exercises at the health centre, heat when it is bad.
I: Do you have an explanation for why it started?
Patricia: I have wondered whether it was a transient blood clot. I was scanned; they considered multiple sclerosis at one point, but luckily that was not it. If you do not arrive at the exact time, maybe you cannot see it afterwards. That is what I lean toward.
I: How do healthcare professionals respond to those thoughts?
Patricia: Mostly the focus returns to treatment. I understand that. But sometimes I am left with the feeling that the cause is not important because the plan is already made. For me, the cause matters because I need the story to make sense.
I: You are part of a walking group after breast cancer. Does that help with dystonia too?
Patricia: Yes, because people who have been ill understand something. It is mostly about cancer, of course, but we can talk about anything. There is a different kind of understanding when someone knows what illness does to ordinary life.
I: You have considered becoming a mentor yourself.
Patricia: Yes, but some people tell me to save my strength. I understand the concern, but I also want to be more than a patient who must conserve energy. Helping someone else can also give energy.

Analytic memo

- Patricia links symptom management to narrative coherence: explanation is part of coping, not an optional add-on.
- Her walking-group account shows how peer understanding may transfer across diagnoses.
- Advice to conserve energy may be protective, but also risks reducing agency.

Candidate codes

pain, self-management, explanatory model, peer support, agency, paternalistic concern

P06. Anne

Profile. Woman, 34 years; disease duration estimated at 16 years; schoolteacher and parent of young children; symptoms began in early adulthood.

Interview setting. Home interview.

Dominant analytic emphasis. Parenting, teaching, fatigue, anticipatory withdrawal, and difficulty obtaining practical support because symptoms fluctuate.

Condensed transcript

I: What is the hardest part of combining dystonia with work and family life?

Anne: The hardest part is that I cannot predict my capacity. In the morning I may think I can teach, pick up the children, cook dinner, and answer emails. By the afternoon, pain and fatigue may make that impossible.

I: How does that affect teaching?

Anne: Teaching is physical in ways people do not notice. You turn toward pupils, write on the board, move around the room, respond quickly. If my head is pulling, I become slower and more irritable because I am trying to keep control.

I: Do colleagues understand?

Anne: They are kind, but kindness is not the same as understanding. Because I can function well some days, it is hard for them to understand why I need accommodations on others. The variability makes me look inconsistent.

I: What about family life?

Anne: Children cannot plan around treatment cycles. They need you when they need you. I feel guilty when I cannot go to activities or when noise and movement become too much. It is not that I do not want to participate; I am calculating whether my neck and my energy will allow it.

I: Have you received rehabilitation or practical guidance?

Anne: Not systematically. I have had injections and some physiotherapy, but no one has sat down with me to map the day: work, children, sleep, pain, transport. That would be more useful than only asking how the last injection worked.

Analytic memo

- Anne makes visible how participation restrictions arise from role accumulation and fluctuating capacity.
- The case expands the corpus toward younger adults and parenting roles, while remaining consistent with the manuscript themes.
- Her account is useful for coding anticipatory withdrawal and the lack of participation-oriented rehabilitation.

Candidate codes

fatigue, parenting, work participation, anticipatory withdrawal, fluctuation, support-system gap

P07. Eva

Profile. Woman, 42 years; disease duration estimated at 13 years; self-employed graphic designer; can work from home but experiences social and leisure restriction.
Interview setting. Cafe interview.
Dominant analytic emphasis. Invisible labour of self-management, home-based work as both accommodation and isolation, online support, and social withdrawal.

Condensed transcript

I: Does working from home make the condition easier to manage?
Eva: It helps practically. I can lie down, use heat, change chairs, or stop for twenty minutes. But it also hides the problem. Because clients only see the finished work, they do not see what it costs.
I: How does that influence social life?
Eva: I cancel more than I want to. The difficult thing is not the cancellation itself; it is the repeated explanation. After a while you stop being invited to spontaneous things because people assume you may not come.
I: Do you talk openly about dystonia?
Eva: Online, yes. In person, less. Online there are people who know the strange details - sensory tricks, bad chairs, how exhausting it is to keep your head still. With friends, I often simplify it to neck pain, because dystonia requires a lecture.
I: What activities have you lost?
Eva: Concerts, cinema, restaurants where the seating is fixed. I still meet people, but I choose places where I can control the chair, noise, and how long I stay. Leisure becomes logistics.
I: What would better support look like?
Eva: A clinician asking: what do you avoid now that you used to enjoy? That question would open a different conversation. I do not only need less muscle activity. I need help getting parts of life back.

Analytic memo

- Eva demonstrates the double-edged nature of home-based adaptation: it preserves productivity while reducing visibility and spontaneous social participation.
- Her language helps operationalise leisure restriction as environmental logistics.

• She supports the integrated interpretation that social participation may be maintained but less spontaneous and less satisfying.

Candidate codes

home-based work, social withdrawal, online support, leisure restriction, environmental adaptation, low satisfaction

P08. Niels

Profile. Man, 47 years; disease duration estimated at 18 years; skilled manual worker; concerned about safety, strength, and being perceived as unreliable.
Interview setting. Cafe interview.
Dominant analytic emphasis. Occupational legitimacy, masculinity, safety, reluctance to request accommodations, and underrecognition by authorities.

Condensed transcript

I: How has cervical dystonia affected your work?
Niels: In manual work, you are expected to be steady. If your head turns or pain distracts you, it can become a safety issue. I can do many tasks, but I need to choose how I position myself. That is not always possible on a site.
I: Do you ask for adjustments?
Niels: Not unless I have to. I do not want to be the man who cannot manage. And because the condition is not well known, adjustments can look like special treatment rather than safety.
I: How do others interpret the symptoms?
Niels: Some think it is stress. Some think it is a bad neck. A few make jokes about looking the wrong way. They do not mean harm, but it tells you they have no idea what it is.
I: What happens when you contact formal support systems?
Niels: You have to explain everything from the beginning. Forms ask whether you can lift, walk, sit, stand. The real answer is: sometimes, depending on pain, posture, and how long I have already been working. The system does not like that answer.
I: What would help you participate more safely?
Niels: A practical assessment at work, not only a diagnosis letter. Someone who understands the task, the posture, the risk, and the fact that I want to keep working. I do not want to be written off; I want the work to be arranged so I can do it.

Analytic memo

- Niels brings occupational risk and legitimacy into the participation framework.
- His reluctance to request accommodation reflects both stigma and gendered expectations of endurance.
- The case illustrates why binary assessments of capacity fail when symptoms fluctuate.

Candidate codes

manual work, occupational safety, masculinity, underrecognition, formal support systems, work accommodation

P09. Thomas

Profile. Man, 49 years; disease duration estimated at 21 years; project manager in IT; long diagnostic pathway; uses formal workplace accommodations but finds healthcare narrowly injection-oriented.
Interview setting. Hospital interview.
Dominant analytic emphasis. Long-term illness career, formal accommodations, cognitive load, treatment-cycle planning, and need for coordinated rehabilitation.

Condensed transcript

I: You have lived with symptoms for many years. What has changed over time?
Thomas: At first the main question was what it was. Later the question became how to build a life around it. I can work, but I organise work around cycles. The good weeks are for demanding meetings; the bad weeks are for tasks I can do with more control.
I: Do formal accommodations help?
Thomas: Yes. Flexible work and home days help. But accommodations also require explanation. Every new manager needs the story. Every new HR form wants a stable limitation. Dystonia is not stable in that way.
I: What is most invisible to others?
Thomas: The cognitive load. Holding your head, managing pain, choosing a chair, thinking about when medication or injections wear off - all of that runs in the background while you are supposed to be concentrating on work.
I: How do you experience specialist care?

Thomas: Competent but narrow. The injections are technically good, and I appreciate that. But there is rarely a plan beyond the next injection. I have had to assemble physiotherapy, ergonomics, and psychological coping myself.

I: What would a more coordinated plan include?

Thomas: A participation review once or twice a year. Work, sleep, pain, exercise, mood, social life. Not a long psychological session necessarily, but a structured conversation that says: this disease affects more than muscles.

Analytic memo

- Thomas provides a longitudinal version of the support-system gap: competence in injection treatment does not equal coordinated care.
- His account supports adding a periodic participation review to clinical assessment.
- The case makes cognitive load a bridge between symptom unpredictability and low satisfaction.

Candidate codes

long-term adaptation, flexible work, cognitive load, symptom-focused care, coordinated rehabilitation, treatment cycle

P10. Mikkel

Profile. Man, 56 years; disease duration estimated at 12 years; previously worked as a driver and amateur musician; now limits driving and public performance because of head position, pain, and embarrassment.

Interview setting. Home interview.

Dominant analytic emphasis. Loss of valued leisure roles, altered identity, family support, physiotherapy, and participation beyond employment.

Condensed transcript

I: What activities do you miss most?

Mikkel: Music and driving without thinking. I used to play with others, not professionally, but enough that it was part of who I was. Now I worry about how I look on stage and whether I can hold the position long enough.

I: Is the worry mainly pain or visibility?

Mikkel: Both. Pain comes first, but visibility follows. If your head pulls, people look. Then you start looking at yourself from the outside. That ruins the pleasure.

I: How has driving changed?

Mikkel: Short trips are fine, especially if the headrest helps. Longer trips are exhausting. I plan routes, breaks, and whether I will have enough energy after arriving. It is not just transport; it decides whether I join family events.

I: How does your family respond?

Mikkel: They are supportive. My wife notices before I admit it. But I do not want every plan to revolve around me. Sometimes I say no early because I do not want to disappoint people later.

I: Have you received rehabilitation support?

Mikkel: Some physiotherapy helped, especially learning how to pace and position myself. But it was not built into the hospital treatment. I had to find it. It would make sense if injections and rehabilitation spoke to each other.

I: What would you like clinicians to ask?

Mikkel: Ask what I have stopped doing. That tells you more than asking only about pain from zero to ten. If I have stopped playing music, that is a treatment issue too.

Analytic memo

- Mikkel highlights leisure participation as identity, not merely recreation.
- His account reinforces that social participation may depend on mobility, energy after arrival, and avoidance of anticipated disappointment.
- He provides a clinically useful formulation: stopped activities should be treated as relevant outcomes.

Candidate codes

leisure identity, driving, family support, anticipatory withdrawal, physiotherapy, participation outcome
